# DNA methylation signatures associated with prognosis of gastric cancer

**DOI:** 10.1186/s12885-021-08389-0

**Published:** 2021-05-25

**Authors:** Jin Dai, Akihiro Nishi, Zhe-Xuan Li, Yang Zhang, Tong Zhou, Wei-Cheng You, Wen-Qing Li, Kai-Feng Pan

**Affiliations:** 1 Key Laboratory of Carcinogenesis and Translational Research (Ministry of Education/Beijing), Department of Cancer Epidemiology, Peking University Cancer Hospital & Institute, 52 Fucheng Rd, Haidian District, Beijing, 100142 People’s Republic of China; 2 Department of Epidemiology, UCLA Fielding School of Public Health, Los Angeles, CA 90095 USA; 3 Joint International Research Center of Translational and Clinical Research, Beijing, 100142 China

**Keywords:** Bioinformatics, Biomarkers, Epigenetics, Gastric cancer, Heterogeneity, Methylation, Precision medicine, Prognosis, Survival, The Cancer Genome Atlas

## Abstract

**Background:**

Few studies have examined prognostic outcomes-associated molecular signatures other than overall survival (OS) for gastric cancer (GC). We aimed to identify DNA methylation biomarkers associated with multiple prognostic outcomes of GC in an epigenome-wide association study.

**Methods:**

Based on the Cancer Genome Atlas (TCGA), DNA methylation loci associated with OS (*n* = 381), disease-specific survival (DSS, *n* = 372), and progression-free interval (PFI, *n* = 383) were discovered in training set subjects (false discovery rates < 0.05) randomly selected for each prognostic outcome and were then validated in remaining subjects (*P*-values < 0.05). Key CpGs simultaneously validated for OS, DSS, and PFI were further assessed for disease-free interval (DFI, *n* = 247). Gene set enrichment analyses were conducted to explore the Gene Ontology and Kyoto Encyclopedia of Genes and Genomes pathways simultaneously enriched for multiple GC prognostic outcomes. Methylation correlated blocks (MCBs) were identified for co-methylation patterns associated with GC prognosis. Based on key CpGs, risk score models were established to predict four prognostic outcomes. Spearman correlation analyses were performed between key CpG sites and their host gene mRNA expression.

**Results:**

We newly identified DNA methylation of seven CpGs significantly associated with OS, DSS, and PFI of GC, including cg10399824 (*GRK5)*, cg05275153 (*RGS12)*, cg24406668 (*MMP9)*, cg14719951(*DSC3)*, and cg25117092 (*MED12L*), and two in intergenic regions (cg11348188 and cg11671115). Except cg10399824 and cg24406668, five of them were also significantly associated with DFI of GC. Neuroactive ligand-receptor interaction pathway was suggested to play a key role in the effect of DNA methylation on GC prognosis. Consistent with individual CpG-level association, three MCBs involving cg11671115, cg14719951, and cg24406668 were significantly associated with multiple prognostic outcomes of GC. Integrating key CpG loci, two risk score models performed well in predicting GC prognosis. Gene body DNA methylation of cg14719951, cg10399824, and cg25117092 was associated with their host gene expression, whereas no significant associations between their host gene expression and four clinical prognostic outcomes of GC were observed.

**Conclusions:**

We newly identified seven CpGs associated with OS, DSS, and PFI of GC, with five of them also associated with DFI, which might inform patient stratification in clinical practices.

**Supplementary Information:**

The online version contains supplementary material available at 10.1186/s12885-021-08389-0.

## Introduction

Gastric cancer (GC) is the fifth most common cancer and third leading cause of cancer deaths worldwide [1]. A considerable proportion of GC patients are diagnosed at advanced stages and their prognosis remains poor [2]. Moreover, GC patients with same clinical stage may develop diverse prognostic outcomes due to the epigenetic or genetic host heterogeneities. Hence, identifying molecular signatures to predict GC prognosis would be warranted for tailored clinical procedures. However, established molecular biomarkers which could predict the prognosis of GC are still very limited.

DNA methylation is a covalent chemical modification, which results in the addition of a methyl (CH_3_) group at the carbon 5 position of the cytosine ring [3]. Alterations in DNA methylation, possessing several merits, such as high frequency in tumor, easy detection, and stability in fixed samples over time, have been considered as promising targets for developing prognostic biomarkers [4]. Based on the Cancer Genome Atlas (TCGA), several studies have identified DNA methylation-based biomarkers associated with overall survival (OS) of GC patients, with three of them based on differentially methylated genes between GC tumor and normal tissue [5–7] and the other one not appropriately account for multiple hypothesis testing [8]. In addition, although OS is the most clinically relevant endpoint with the least equivocal definition, solely using OS as the prognostic endpoint may not be sufficient to mirror all perspectives of tumor prognosis, as patient deaths may be due to GC or other causes [9]. Disease-specific survival (DSS) therefore would possess greater relevance to GC-specific biology and therapeutic impact. In addition, given the relatively short follow-up period of GC (median follow-up time = 14 months) in TCGA, the number of deaths at the end of follow-up may be insufficient to reveal a difference in deaths among comparison groups [10, 11]. Progression-free interval (PFI) and disease-free interval (DFI) have therefore been considered as better choices of surrogate clinical endpoints. To our best knowledge, few studies have identified DNA methylation-based biomarkers associated with prognostic outcomes other than OS of GC patients.

In the present epigenome-wide association study, we comprehensively examined the association between DNA methylation and four prognostic outcomes of GC, including OS, DSS, PFI, and DFI of GC patients in TCGA.

## Materials and methods

### Data sources and preprocessing

DNA methylation profile (IDAT files) from 395 GC patients were obtained using R/Bioconductor package TCGAbiolinks [12]. In TCGA, DNA methylation of cancer tissue from all patients were assayed using the Infinium HumanMethylation450 BeadChip arrays. A TCGA online tool was used to estimate batch effect (https://bioinformatics.mdanderson.org/BatchEffectsViewer/), which would be presented if the dispersion separability criterion (DSC) value was greater than 0.5 and the DSC *P*-value was less than 0.05. Data importation, annotation, and quality control were conducted in the R/Bioconductor package minfi [13]. We used R/Bioconductor package SeSAMe to perform background correction and dye-bias correction [14, 15]. In brief, normal-exponential using out-of-band probes (NOOB) background correction was utilized to account for additive error to the measure of signal intensities due to background fluorescence. Non-linear dye-bias correction was performed to control for the different average intensities in the red and green channels of Type II probes. In addition, *P*-value with out-of-band (OOB) array hybridization (POOBAH) method was applied to address artifacts which reflected failed hybridization to target DNA respectively. For probe filtering, we excluded probes for which the detection *P*-value was bigger than 0.05 for more than 10% of the remaining samples, those on the sex chromosomes, those mapped to multiple regions in the genome, and those harboring known single-nucleotide polymorphisms with a minor allele frequency greater than 5% [16]. After data preprocessing, a total of 411,408 CpG sites were kept in our analysis, including 163,453 in promoter, 139,213 in gene body, 14,896 in 3’UTR, and 93,846 in intergenic regions.

Demographical and clinical characteristics of GC patients, including age at initial pathologic diagnosis, gender, ethnicity, histological grade, anatomic region, family history of GC, *Helicobacter pylori* infection, radiation therapy, and the tumor, lymph nodes, and metastasis (TNM) stage were obtained using R/Bioconductor package TCGAbiolinks [12]. We further imputed missing ethnicities using R/Bioconductor package SeSAMe [15]. Four prognostic outcomes, including OS, DSS, PFI, and DFI of GC, were derived from those defined in a previous study [11]. In short, using the date of diagnosis of primary GC as the beginning date, OS was defined as the period until the date of death from any cause; DSS was defined as the period until the date of GC-specific death; PFI was defined as the period until the date of the first occurrence of a new GC; DFI was defined as the period until the date of the first new GC progression event subsequent to the ascertainment of a patient’s disease-free status after their initial GC diagnosis and treatment. The GC-free status of patients must be confirmed in order for them to be qualified for calculating DFI.

### Identification of key CpG sites associated with four clinical endpoints

For all 411,408 CpG sites, we first examined the association between DNA methylation of each individual CpG site and OS of GC. GC patients with information on OS were randomly divided into training and validation sets (50:50). For CpG sites significantly associated with OS in the training set (false discovery rate (FDR) < 0.05 considering multiple comparisons) [17], we sought to replicate the associations in the validation sets (*P* < 0.05). For those replicated CpG loci, associations with DSS and PFI of GC were then examined. GC patients with information on DSS or PFI were randomly divided into training and validation sets (50:50) for the assessment of each specific outcome. We then selected key CpG sites significantly associated with OS, DSS, and PFI in the respective training and validation sets, which were further assessed for the association with DFI. Due to the difficulty of ascertaining GC-free status of patients after their initial diagnosis and treatment, a large body of patients, especially for those in stage IV, were excluded in DFI analysis. We therefore did not seek to divide it into training and validation sets for statistical power consideration. Cox proportional hazard analyses were performed to calculate hazard ratios (HRs) and 95% confidence intervals (CIs) for the association between each individual CpG site and clinical endpoint, adjusting for age at initial pathologic diagnosis (continuous) and gender. For those CpG sites significantly associated with clinical endpoints, sensitivity analyses were conducted to test the robustness of the associations by additionally adjusting for other potential confounders, including ethnicity (Asian, Black or African American, or White), TNM stage (T1–2, T3–4, or unknown; N0–1, N2–3, or unknown; M0, M1, or unknown), histological grade (G1–2, G3, or unknown), anatomic neoplasm region (antrum/distal, cardia/proximal, fundus/body, gastroesophageal junction, or unknown), radiation therapy (yes, no, or unknown), *H. pylori* infection (yes, no, or unknown), and family history of GC (yes, no, or unknown). We did not find violations of proportional hazard assumptions for all Cox proportional hazard models.

### Gene set enrichment analysis

Gene set enrichment analyses (GSEA) were conducted to explore the Gene Ontology (GO) and Kyoto Encyclopedia of Genes and Genomes (KEGG) pathways which simultaneously enriched for multiple GC prognostic outcomes using DNA methylation data [18, 19]. The analyses were conducted utilizing functional class scoring approach, which overcomes the drawbacks of over-representation analysis, another commonly used method for GSEA [20]. As most of the CpG sites significantly associated with GC prognosis in our study were in gene body, we focused on CpG sites located in gene body region only for GSEA. All preprocessed CpG sites in gene body region were ranked based on their Cox regression *P*-values with GC prognosis. Gene set level *P*-values were computed through permutation using R/Bioconductor package methylGSA [21]. The number of CpG sites located in one gene was adjusted in the logistic model to avoid length bias. Gene sets were defined as those containing at least 10 genes and at most 500 genes. FDR less than 0.05 was set as the criteria defining significantly enriched GO or KEGG terms.

### Identification of methylation correlated blocks (MCBs) based on key CpGs

In addition to individual CpG loci-level analyses, we also examined the association for a cluster of CpG sites located in the same genomic region based on the concept of genetic linkage disequilibrium. To investigate whether key CpG sites had co-methylation patterns with their adjacent CpG sites and whether these patterns were associated with GC prognosis, Pearson correlation analyses were conducted between DNA methylation level of key CpG sites and their adjacent loci positioned within their flanking areas of one kilobase up- or downstream. Pearson correlation coefficients less than 0.5 indicated boundaries of uncorrelated methylation [22]. For adjacent loci that were not separated by boundaries, we continued examining the correlation between DNA methylation level of these loci and their neighboring loci located within their flanking areas. All CpG sites not separated by the boundary were combined into methylation correlated blocks (MCBs) [22]. The mean *β* values of all CpG sites within one MCB was defined as the methylation value of that MCB. The R/Bioconductor co-MET package was utilized to visualize the co-methylation patterns of identified MCBs [23]. We then examined the association between those identified MCBs and four clinical endpoints using Cox regression analyses adjusting for age at initial pathologic diagnosis (continuous) and gender. For significant MCBs, sensitivity analyses were also performed, similar to those conducted for individual CpG sites.

### Establishment of two risk score models

Incorporating key CpG sites associated with GC prognosis, risk score models to predict the risk of specific prognostic outcomes were constructed, which were defined as the sum of auto-scaled DNA methylation level of each CpG weighted by the regression coefficients obtained from the Cox regression model in the whole dataset:

$$ \mathrm{Risk}\ \mathrm{score}={\upbeta}_1\ {\mathrm{X}}_1+{\upbeta}_2{\mathrm{X}}_2+{\upbeta}_3\ {\mathrm{X}}_3+\dots \dots +{\upbeta}_{\mathrm{n}}\ {\mathrm{X}}_{\mathrm{n}}, $$

GC patients were categorized into low-, median-, and high- risk score groups based on the tertile of their risk scores. Kaplan-Meier survival curves were drawn and log-rank tests were performed to compare the survival probabilities among different risk score groups.

### Correlation between DNA methylation and mRNA expression

To explore the potential regulating effect of DNA methylation on host gene expression, Spearman correlation analyses were performed between DNA methylation of those key CpG sites and their host gene mRNA expression. Normalized encoding genes’ mRNA expression data (legacy data) based on RNA-Seq (Illumina RNA-Seq HiSeq platform) was downloaded and preprocessed using R/Bioconductor package TCGAbiolinks [12]. Both mRNA expression level and DNA methylation level (*β* value) were auto-scaled before down-stream analysis. *P*-value < 0.05 was set as the criteria for the significant correlation. For those genes whose DNA methylation was correlated with their mRNA expression, the association of their mRNA expression with four clinical endpoints of GC were then examined using Cox regression analysis.

All analyses were performed and visualized using R version 4.0.2.

## Results

### Major characteristics of GC patients in TCGA

In the present study, we included GC patients having both DNA methylation profiles and intact follow-up information of OS (*n* = 381), DSS (*n* = 372), PFI (*n* = 383), and DFI (*n* = 247). During the follow-up, 149, 99, 137, and 46 GC patients developed corresponding clinical endpoints of OS (death from any cause), DSS (GC-specific death), PFI (the first occurrence of a new GC event), and DFI (the first new GC progression event), respectively. Major characteristics of included GC patients were shown in Table 1.

**Table 1:** Major Characteristics of GC patients with multiple clinical prognostic endpoints in TCGA

	OS		DSS		PFI		DFI
	Training	Validation	Training	Validation	Training	Validation	
**Total number, No**	191	190	186	186	192	191	247
**Ethnicity, No. (%)**^**a**^							
**Asian**	42 (22.0)	41 (21.6)	38 (20.4)	42 (22.6)	46 (24.0)	37 (19.4)	62 (25.1)
**Black or African American**	9 (4.7)	5 (2.6)	6 (3.2)	8 (4.3)	10 (5.2)	5 (2.6)	7 (2.8)
**White**	140 (73.3)	144 (75.8)	142 (76.3)	136 (73.1)	136 (70.8)	149 (78.0)	178 (72.1)
**Age, mean (SD)**	28.9 (10.7)	29.2 (10.3)	29.1 (10.9)	28.7 (10.1)	29.1 (10.5)	29.0 (10.5)	29. 3 (10.6)
**Gender, No. (%)**							
**Male**	125 (65.4)	126 (66.3)	123 (66.1)	121 (65.1)	133 (69.3)	120 (62.8)	152 (61.5)
**Female**	66 (34.6)	64 (33.7)	63 (33.9)	65 (34.9)	59 (30.7)	64 (37.2)	95 (38.5)
**Clinical stage, No. (%)**							
**Stage I**	21 (11.0)	27 (14.2)	27 (14.5)	20 (10.8)	29 (15.1)	20 (10.5)	40 (16.2)
**Stage II**	65 (34.0)	56 (29.5)	60 (32.3)	58 (31.2)	61 (31.8)	60 (31.4)	97 (39.3)
**Stage III**	85 (44.5)	85 (44.7)	77 (41.4)	88 (47.3)	80 (41.7)	91 (47.6)	106 (42.9)
**Stage IV**	16 (8.4)	15 (7.9)	14 (7.5)	17 (9.1)	18 (9.4)	13 (6.8)	–
**Unknown**	4 (2.1)	7 (3.7)	8 (4.3)	3 (1.6)	4 (2.1)	7 (3.7)	4 (1.6)
**Histologic grade, No. (%)**							
**G1–2**	67 (35.1)	75 (39.5)	76 (40.9)	63 (33.9)	73 (38.0)	70 (36.6)	91 (36.8)
**G3**	120 (62.8)	110 (57.9)	106 (57.0)	118 (63.4)	113 (58.9)	118 (61.8)	151 (61.1)
**Unknown**	4 (2.1)	5 (2.6)	4 (2.2)	5 (2.7)	6 (3.1)	3 (1.6)	5 (2.0)
**Radiation therapy, No. (%)**							
**Yes**	29 (15.2)	21 (11.1)	19 (10.2)	31 (16.7)	30 (15.6)	22 (11.5)	40 (16.2)
**No**	127 (66.5)	123 (64.7)	129 (69.4)	120 (64.5)	119 (62.0)	131 (68.6)	187 (75.7)
**Unknown**	35 (18.3)	46 (24.2)	38 (20.4)	35 (18.8)	43 (22.4)	38 (19.9)	20 (8.1)
***H. pylori*** **infection, No. (%)**							
**Yes**	11 (5.8)	9 (4.7)	9 (4.8)	11 (5.9)	7 (3.6)	13 (6.8)	15 (6.1)
**No**	84 (44.0)	80 (42.1)	79 (42.5)	80 (43.0)	90 (46.9)	75 (39.3)	93 (37.7)
**Unknown**	96 (50.3)	101 (53.2)	98 (52.7)	95 (51.1)	95 (49.5)	103 (53.9)	139 (56.3)
**Family history of GC, No. (%)**							
**Yes**	11 (5.8)	6 (3.2)	8 (4.3)	9 (4.8)	8 (4.2)	10 (5.2)	11 (4.5)
**No**	158 (82.7)	157 (82.6)	155 (83.3)	154 (82.8)	162 (84.4)	153 (80.1)	203 (82.2)
**Unknown**	22 (11.5)	27 (14.2)	23 (12.4)	23 (12.4)	22 (11.5)	28 (14.7)	33 (13.4)
**Anatomic subdivision (%)**							
**Antrum/Distal**	77 (40.3)	64 (33.7)	65 (34.9)	71 (38.2)	71 (37.0)	70 (36.6)	92 (37.2)
**Cardia/Proximal**	24 (12.6)	28 (14.7)	34 (18.3)	18 (9.7)	27 (14.1)	25 (13.1)	28 (11.3)
**Fundus/Body**	66 (34.6)	72 (37.9)	59 (31.7)	76 (40.9)	70 (36.5)	68 (35.6)	99 (40.1)
**Gastroesophageal Junction**	19 (9.9)	22 (11.6)	21 (11.3)	19 (10.2)	19 (9.9)	23 (12.0)	23 (9.3)
**Unknown**	5 (2.6)	4 (2.1)	7 (3.8)	2 (1.1)	5 (2.6)	5 (2.6)	5 (2.0)
**Pathologic T, No. (%)**							
**T1–2**	44 (23.0)	48 (25.3)	52 (28.0)	38 (20.4)	49 (25.5)	44 (23.0)	63 (25.5)
**T3–4**	147 (77.0)	142 (74.7)	134 (72.0)	148 (79.6)	143 (74.5)	147 (77.0)	184 (74.5)
**Pathologic N, No. (%)**							
**N0–1**	114 (59.7)	103 (54.2)	111 (59.7)	99 (53.2)	109 (56.8)	109 (57.1)	164 (66.4)
**N2–3**	74 (38.7)	82 (43.2)	70 (37.6)	84 (45.2)	81 (42.2)	76 (39.8)	78 (31.6)
**Unknown**	3 (1.6)	5 (2.6)	5 (2.7)	3 (1.6)	2 (1.0)	6 (3.1)	5 (2.0)
**Pathologic M, No. (%)**							
**M0**	173 (90.6)	169 (88.9)	166 (89.2)	169 (90.9)	171 (89.1)	172 (90.1)	238 (96.4)
**M1**	9 (4.7)	13 (6.8)	12 (6.5)	10 (5.4)	11 (5.7)	11 (5.8)	–
**Unknown**	9 (4.7)	8 (4.2)	8 (4.3)	7 (3.8)	10 (5.2)	8 (4.2)	9 (3.6)
**Prognostic endpoints, No. (%)**							
**Event**	77 (40.3)	72 (37.9)	55 (29.6)	44 (23.7)	77 (40.1)	60 (31.4)	46 (18.6)
**Censored**	114 (59.7)	118 (62.1)	131 (70.4)	142 (76.3)	115 (59.9)	131 (68.6)	201 (81.4)

*Abbreviations*: *GC* gastric cancer, *OS* overall survival, *DSS* disease-specific survival, *DFI* disease-free interval, *PFI* progression-free interval

^a^Missing values of ethnicity were imputed using R package SeSAMe

### Identification of key CpG sites associated with four clinical endpoints

According to TCGA Batch Effect Viewer, no significant batch effect was observed (DSC = 0.307, DSC *P*-value = 5 × 10^−4^). After data preprocessing, 411,408 CpG sites were kept in our analysis. By performing the Cox regression analyses for all preprocessed CpGs, we identified a total of 488 CpG sites significantly associated with OS (FDR < 0.05 in the training set and *P*-value < 0.05 in the validation set). Among them, seven key CpG sites were simultaneously associated with DSS and PFI in both training (FDR < 0.05) and validation sets (*P*-value < 0.05), including cg10399824 (G protein-coupled receptor kinase 5, *GRK5*), cg05275153 (regulator of G protein signaling 12, *RGS12*), cg24406668 (matrix metallopeptidase 9, *MMP9*), cg14719951 (desmocollin 3, *DSC3*), and cg25117092 (mediator complex subunit 12 L, *MED12L/MED12*) and two CpG loci (cg11348188 and cg11671115) located within intergenic regions. Of them, DNA hypermethylation of three CpG sites, including cg11348188 and cg11671115 in intergenic regions, and cg25117092 in gene body was significantly associated with unfavorable OS, DSS, and PFI of GC, whereas DNA hypermethylation of cg05275153, cg14719951, cg10399824, and cg24406668 in gene body was significantly associated with favorable OS, DSS, and PFI of GC. These CpG loci were further assessed for DFI of GC. Except for cg10399824 and cg24406668, DNA methylation level of the other five CpG sites was also significantly associated with DFI. The directions of associations between seven key CpG sites and all four clinical endpoints remained consistent (Table 2).

**Table 2:** The hazard ratios and *P*-values for the associations between DNA methylation of seven key CpG loci and four clinical prognostic endpoints of GC patients in TCGA

CpG sites	Genomic positions	Gene names	Genomic groups (CpG contents)	Training set			Validation set		Pooled set	
				HR (95% CI)^**a**^	***P***-value	FDR	HR (95% CI)^**a**^	***P***-value	HR (95% CI)^**a**^	***P***-value
**OS**										
**cg11348188**	chr2–222,655,366	*EPHA4, PAX3*	Intergenic (open sea)	1.69 (1.32–2.18)	3.84 × 10^−5^	0.012	1.33 (1.05–1.68)	0.017	1.46 (1.23–1.72)	1.44 × 10^− 5^
**cg25117092**^**a**^	chr3–150,948,274	*MED12L*	Body (open sea)	1.67 (1.29–2.16)	1.03 × 10^−4^	0.017	1.28 (1.01–1.62)	0.037	1.42 (1.20–1.69)	4.70 × 10^− 5^
**cg05275153**^**b**^	chr4–3,431,837	*RGS12*	Body (open sea)	0.79 (0.68–0.91)	9.97 × 10^− 4^	0.042	0.69 (0.56–0.86)	8.99 × 10^− 4^	0.74 (0.65–0.84)	3.02 × 10^−6^
**cg11671115**	chr10–87,336,486	*FAM190B, GRID1*	Intergenic (open sea)	1.58 (1.22–2.04)	4.85 × 10^− 4^	0.032	1.36 (1.08–1.71)	0.010	1.42 (1.20–1.67)	3.52 × 10^−5^
**cg14719951**^**c**^	chr18–28,622,474	*DSC3*	Body (island)	0.69 (0.55–0.87)	1.33 × 10^−3^	0.048	0.74 (0.58–0.93)	0.010	0.72 (0.61–0.84)	4.22 × 10^−5^
**cg10399824**^**d**^	chr10–121,048,354	*GRK5*	Body (open sea)	0.61 (0.50–0.75)	3.67 × 10^−6^	0.005	0.65 (0.53–0.80)	4.01 × 10^−5^	0.65 (0.56–0.74)	7.00 × 10^−10^
**cg24406668**^**e**^	chr20–44,639,431	*MMP9*	Body (island)	0.59 (0.46–0.76)	2.88 × 10^−5^	0.011	0.78 (0.62–0.99)	0.037	0.70 (0.59–0.82)	2.29 × 10^−5^
**DSS**										
**cg11348188**	chr2–222,655,366	*EPHA4, PAX3*	Intergenic (open sea)	1.54 (1.16–2.06)	3.10 × 10^−3^	0.028	2.05 (1.45–2.90)	4.41 × 10^−5^	1.73 (1.39–2.15)	9.42 × 10^−7^
**cg25117092**^**a**^	chr3–150,948,274	*MED12L*	Body (open sea)	1.49 (1.10–2.00)	8.95 × 10^−3^	0.043	1.75 (1.27–2.40)	5.66 × 10^−4^	1.60 (1.29–1.98)	2.17 × 10^−5^
**cg05275153**^**b**^	chr4–3,431,837	*RGS12*	Body (open sea)	0.67 (0.55–0.82)	1.04 × 10^−4^	0.005	0.70 (0.53–0.91)	0.009	0.69 (0.59–0.80)	1.32 × 10^−6^
**cg11671115**	chr10–87,336,486	*FAM190B, GRID1*	Intergenic (open sea)	1.59 (1.19–2.12)	1.79 × 10^−3^	0.020	1.71 (1.26–2.31)	5.65 × 10^−4^	1.65 (1.34–2.04)	2.43 × 10^−6^
**cg14719951**^**c**^	chr18–28,622,474	*DSC3*	Body (island)	0.64 (0.49–0.85)	1.62 × 10^−3^	0.020	0.65 (0.49–0.88)	0.005	0.65 (0.53–0.79)	2.16 × 10^−5^
**cg10399824**^**d**^	chr10–121,048,354	*GRK5*	Body (open sea)	0.54 (0.42–0.69)	9.20 × 10^−7^	1.31 × 10^−4^	0.57 (0.44–0.73)	7.12 × 10^−6^	0.57 (0.48–0.67)	1.65 × 10^−11^
**cg24406668**^**e**^	chr20–44,639,431	*MMP9*	Body (island)	0.71 (0.53–0.93)	1.46 × 10^−2^	0.050	0.54 (0.39–0.74)	1.49 × 10^− 4^	0.62 (0.50–0.76)	6.56 × 10^−6^
**PFI**										
**cg11348188**	chr2–222,655,366	*EPHA4, PAX3*	Intergenic (open sea)	1.65 (1.27–2.13)	1.53 × 10^−4^	0.021	1.53 (1.17–1.99)	0.002	1.58 (1.32–1.89)	7.00 × 10^−7^
**cg25117092**^**a**^	chr3–150,948,274	*MED12L*	Body (open sea)	1.47 (1.16–1.86)	1.53 × 10^−3^	0.049	1.48 (1.12–1.94)	0.005	1.49 (1.25–1.79)	1.07 × 10^−5^
**cg05275153**^**b**^	chr4–3,431,837	*RGS12*	Body (open sea)	0.71 (0.59–0.87)	6.84 × 10^−4^	0.043	0.54 (0.41–0.72)	2.11 × 10^−5^	0.70 (0.61–0.80)	2.17 × 10^−7^
**cg11671115**	chr10–87,336,486	*FAM190B, GRID1*	Intergenic (open sea)	1.57 (1.24–1.99)	1.86 × 10^−4^	0.021	1.40 (1.08–1.83)	0.011	1.51 (1.27–1.80)	3.12 × 10^−6^
**cg14719951**^**c**^	chr18–28,622,474	*DSC3*	Body (island)	0.69 (0.55–0.87)	1.52 × 10^−3^	0.049	0.77 (0.60–0.99)	0.043	0.73 (0.62–0.86)	2.48 × 10^−4^
**cg10399824**^**d**^	chr10–121,048,354	*GRK5*	Body (open sea)	0.57 (0.46–0.7)	1.26 × 10^−7^	5.62 × 10^−5^	0.75 (0.59–0.96)	0.020	0.63 (0.54–0.74)	5.10 × 10^−9^
**cg24406668**^**e**^	chr20–44,639,431	*MMP9*	Body (island)	0.68 (0.54–0.86)	1.24 × 10^−3^	0.049	0.68 (0.52–0.90)	0.007	0.69 (0.58–0.83)	4.74 × 10^−5^
**DFI**										
**cg11348188**	chr2–222,655,366	*EPHA4, PAX3*	Intergenic (open sea)	–	–	–	–	–	1.51 (1.12–2.05)	0.007
**cg25117092**^**a**^	chr3–150,948,274	*MED12L*	Body (open sea)	–	–	–	–	–	1.75 (1.27–2.42)	0.001
**cg05275153**^**b**^	chr4–3,431,837	*RGS12*	Body (open sea)	–	–	–	–	–	0.77 (0.63–0.94)	0.012
**cg11671115**	chr10–87,336,486	*FAM190B, GRID1*	Intergenic (open sea)	–	–	–	–	–	1.59 (1.18–2.14)	0.002
**cg14719951**^**c**^	chr18–28,622,474	*DSC3*	Body (island)	–	–	–	–	–	0.74 (0.56–0.99)	0.042
**cg10399824**^**4**^	chr10–121,048,354	*GRK5*	Body (open sea)	–	–	–	–	–	0.78 (0.60–1.01)	0.059
**cg24406668**^**5**^	chr20–44,639,431	*MMP9*	Body (island)	–	–	–	–	–	0.77 (0.57–1.04)	0.089

*Abbreviations*: *GC* gastric cancer, *OS* overall survival, *DSS* disease-specific survival, *DFI* disease-free interval, *PFI* progression-free interval, *HRs* hazard ratios, *CI* confidence interval, *FDR* false discovery rate

^a^Cox regression analyses adjusting for age at initial pathologic diagnosis (continuous) and gender (male, female).

^b^Distance to the closest TSS for cg25117092 (MED12L) is 39,570 bp

^c^Distance to the closest TSS for cg05275153 (RGS12) is 2021 bp

^d^Distance to thee closest TSS for cg14719951 (DSC3) is 308 bp

^e^Distance to the closest TSS for cg10399824 is (GRK5) 81271 bp

^f^Distance to the closest TSS for cg24406668 is (MMP9) 1884 bp

### GSEA based on CpGs within gene body

Since five key CpG sites that had their host gene information were all located in gene body, GSEA was conducted exclusively based on all CpG sites located in gene body to elucidate the biological mechanisms of gene body DNA methylation on GC prognosis. A total of five GO terms were enriched in all four clinical endpoints according to their original *P*-values, but none remained simultaneously significant for all four endpoints after multiple comparison adjustment. Four KEGG pathways were simultaneously enriched in OS, DSS, and PFI, with one pathway involving neuroactive ligand-receptor interaction remaining consistently significant even after multiple comparison adjustment (Table 3, Supplementary Table S1).

**Table 3:** Gene set enrichment analysis for gene body DNA methylation in relation to four clinical endpoints of GC patients in TCGA

ID	Description	Number of genes	Number of unique genes	OS		DSS		PFI		DFI	
				*P*-value	FDR	*P*-value	FDR	*P*-value	FDR	*P*-value	FDR
GO^a^											
35267	NuA4 histone acetyltransferase complex	31	20	0.002	0.066	0.007	0.197	0.047	0.477	0.002	0.042
36019	Endolysosome	21	20	0.012	0.219	0.002	0.078	0.006	0.152	0.017	0.220
36020	Endolysosome membrane	14	14	0.007	0.154	0.001	0.036	0.003	0.106	0.036	0.364
43189	H4/H2A histone acetyltransferase complex	31	20	0.002	0.066	0.007	0.197	0.047	0.477	0.002	0.042
44346	Fibroblast apoptotic process	24	24	0.036	0.409	0.024	0.409	0.029	0.370	1.51 × 10^−4^	0.008
KEGG											
04080	Neuroactive ligand-receptor interaction	272	272	1.24 × 10^−16^	2.64 × 10^−14^	2.32 × 10^−16^	4.95 × 10^−14^	3.44 × 10^−10^	7.34 × 10^−8^	1.000	1.000
04144	Endocytosis	201	201	0.005	0.324	0.026	0.923	0.027	0.645	0.194	0.858
04950	Maturity onset diabetes of the young	25	25	0.037	0.901	0.010	0.634	0.008	0.329	1.000	1.000
05217	Basal cell carcinoma	55	55	0.017	0.712	0.011	0.634	0.035	0.749	1.000	1.000

*Abbreviations*: *GC* gastric cancer, *OS* overall survival, *DSS* disease-specific survival, *DFI* disease-free interval, *PFI* progression-free interval, *HRs* hazard ratios, *CI* confidence interval, *FDR* false discovery rate

^a^Each GO annotation includes an evidence code to indicate how the annotation to a particular term is supported. If a gene is linked to a GO annotation by two distinct evidence codes (e.g., Inferred from Direct Assay (IDA) and Inferred from Biological aspect of Ancestor (IBA)), it would be counted twice to the number of genes enriched in that gene set

### Identification of MCBs associated with GC prognosis

**Fig. 1:**
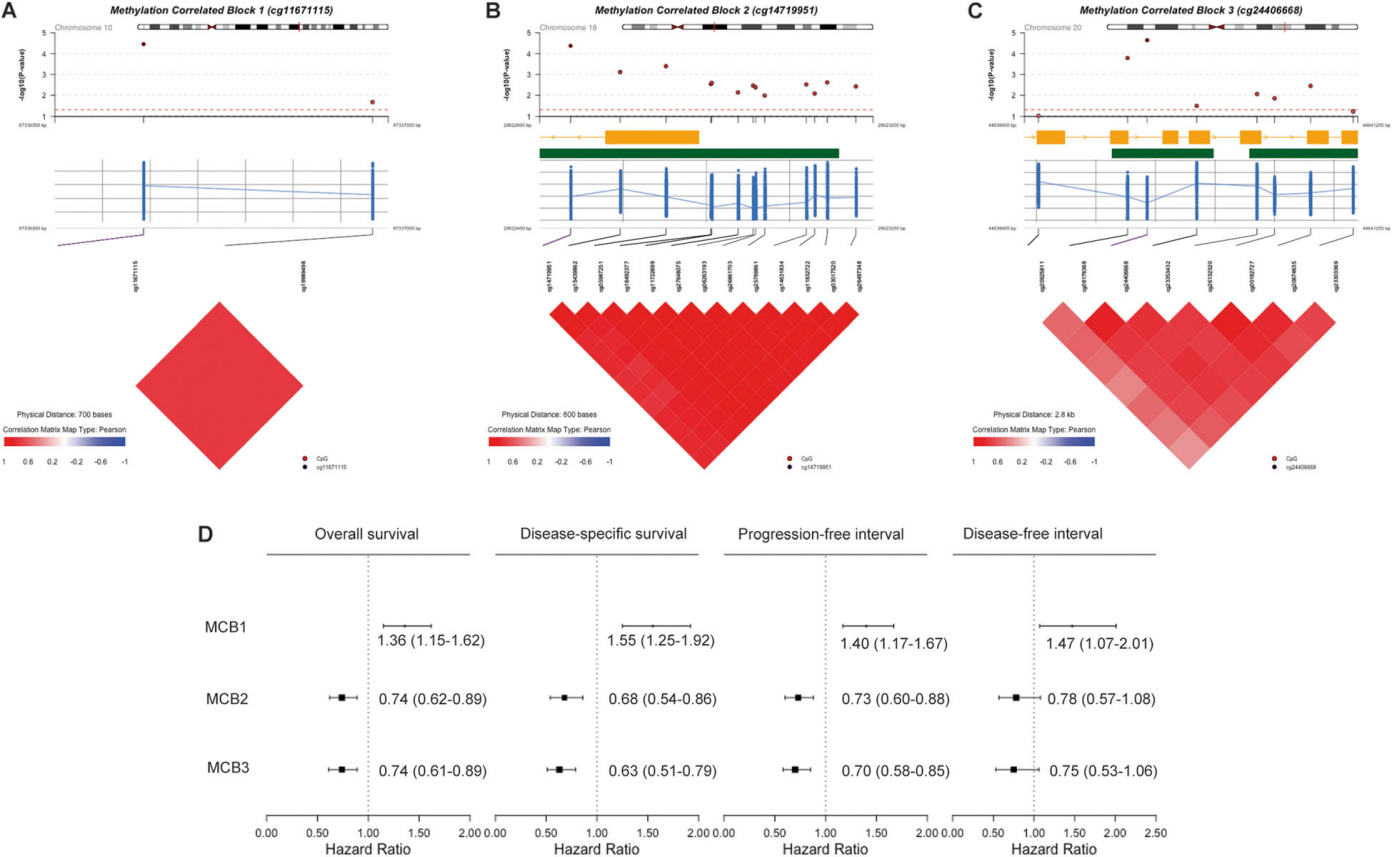
Methylation correlated blocks (MCBs). **a** MCBs based on cg11671115 and adjacent CpG sites in chromosome 10. **b** MCBs based on cg14719951 and adjacent CpG sites in chromosome 18. **c** MCBs based on cg24406668 and adjacent CpG sites in chromosome 20. **d** Forest plots indicating the association between DNA methylation of three MCBs and four clinical endpoints. For (**a**), (**b**), and (**c**), the circles indicate *P*-values of the associations between each CpG within the MCBs and OS. The red dotted lines represent the significant level (*P* = 0.05). The yellow bars represent locations of genes and the green bars represent locations of CpG islands. The blue dots represent DNA methylation level of each CpG within the MCBs. The blue lines connect mean DNA methylation level of each CpG within the MCBs

Investigating the potential co-methylation patterns focusing on seven key CpG sites, we identified three MCBs, including one consisting of cg11671115 and cg19989498 in the intergenic region, another containing cg14719951 and 12 adjacent CpG sites in *DSC3* gene body, and the third one including cg24406668 and seven adjacent CpG sites in *MMP9* gene body. Consistent with individual CpG-level association, DNA methylation of these three MCBs was significantly associated with multiple prognostic outcomes of GC (Fig. 1).

### Establishment of two risk score models

Based on DNA methylation of the seven key CpG sites associated with OS, DSS, and PFI of GC, a risk score models was established:

$$ \mathrm{Model}\ 1=\left(-0.301\right)\mathrm{cg}05275153+\left(-0.436\right)\ \mathrm{cg}10399824+(0.375)\mathrm{cg}11348188+(0.348)\mathrm{cg}11671115+\left(-0.335\right)\mathrm{cg}14719951+\left(-0.363\right)\mathrm{cg}24406668+(0.353)\mathrm{cg}25117092 $$

We also established a risk score model based on the five CpG sites associated with DFI:

$$ \mathrm{Model}\ 2=\left(-0.301\right)\mathrm{cg}05275153+(0.375)\mathrm{cg}11348188+(0.348)\mathrm{cg}11671115+\left(-0.335\right)\ \mathrm{cg}14719951+(0.353)\mathrm{cg}25117092 $$

**Fig. 2:**
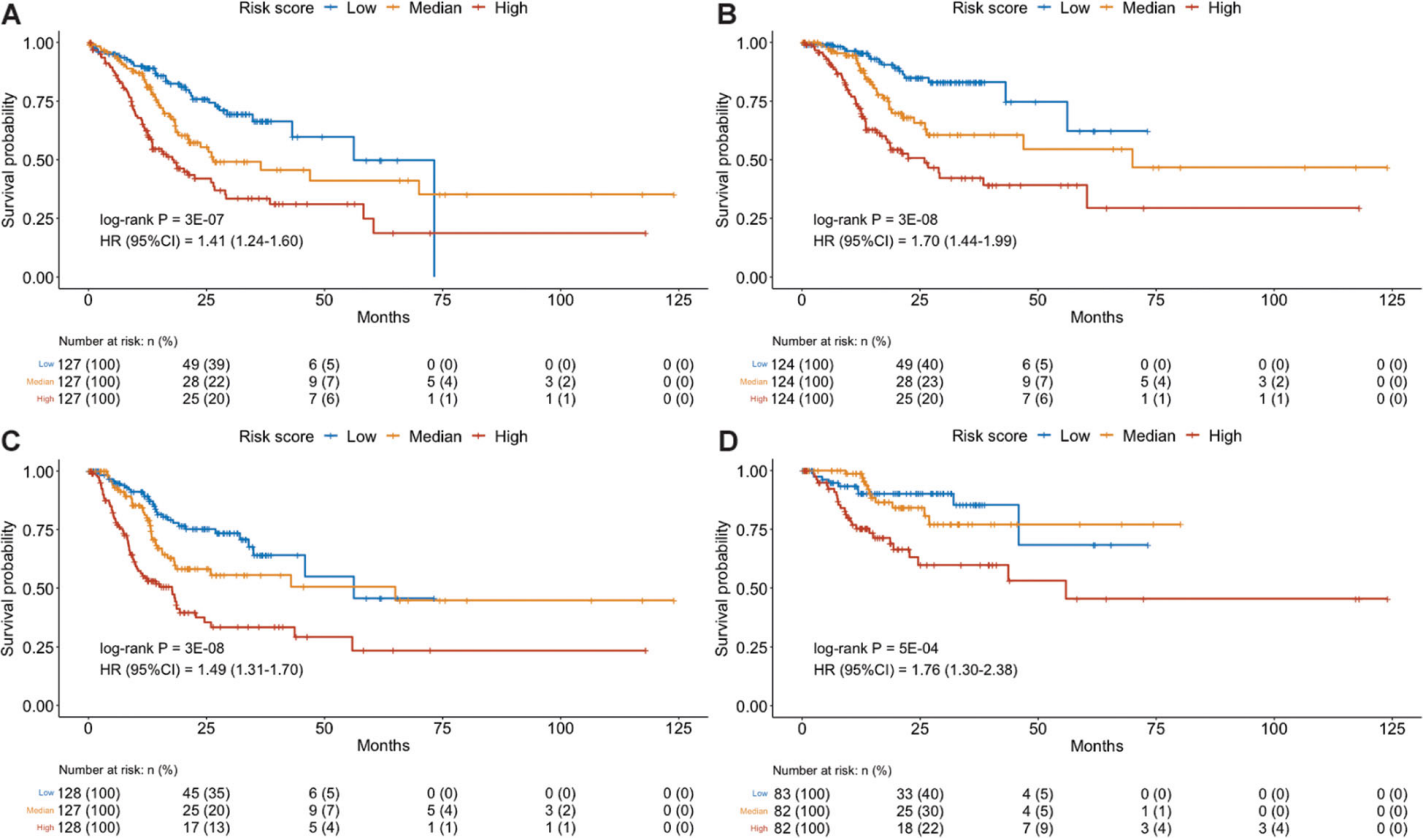
Kaplan-Meier (KM) survival curves of the DNA methylation-based risk score models in TCGA. **a** The seven-CpG based risk score model in OS dataset. **b** The seven-CpG based risk score model in DSS dataset. **c** The seven-CpG based risk score model in PFI dataset. **D** The five-CpG based risk score model in DFI dataset. In each dataset, GC patients were equally categorized into three groups based on the tertile of risk scores of all patients. Blue curves represent low risk score groups; Yellow curves represent median risk score groups; Red curves represent high risk score groups. Log-rank tests were used to compare survival curves among subgroups for each prognostic outcome of GC

For both models, higher scores indicated worse prognosis potential. After adjusting for all available, potential confounders, both risk scores were independently associated with corresponding clinical endpoints, with a HR (95% CI) per one score increase of 1.41 (1.24–1.60) for OS, 1.70 (1.44–1.99) for DSS, 1.49 (1.31–1.70) for PFI, and 1.76 (1.30–2.38) for DFI. Kaplan-Meier survival curves showed that two models performed well on predicting OS (log-rank *P* = 3 ×10^−7^), DSS (*P* = 3 ×10^−8^), PFI (*P* = 3 ×10^−8^), and DFI (*P* = 5 ×10^−4^) (Fig. 2). Integrating with all clinicopathologic features, our risk score models achieved the Harrell’s concordance index (C-index) of a mean (standard deviation (SD)) of 0.770 (0.019) for OS, 0.794 (0.021) for DSS, 0.743 (0.023) for PFI, and 0.784 (0.030) for DFI, which outperformed in prediction compared with models solely utilizing either clinicopathologic features or DNA methylation signatures.

### Correlation analysis between DNA methylation and mRNA expression

**Fig. 3:**
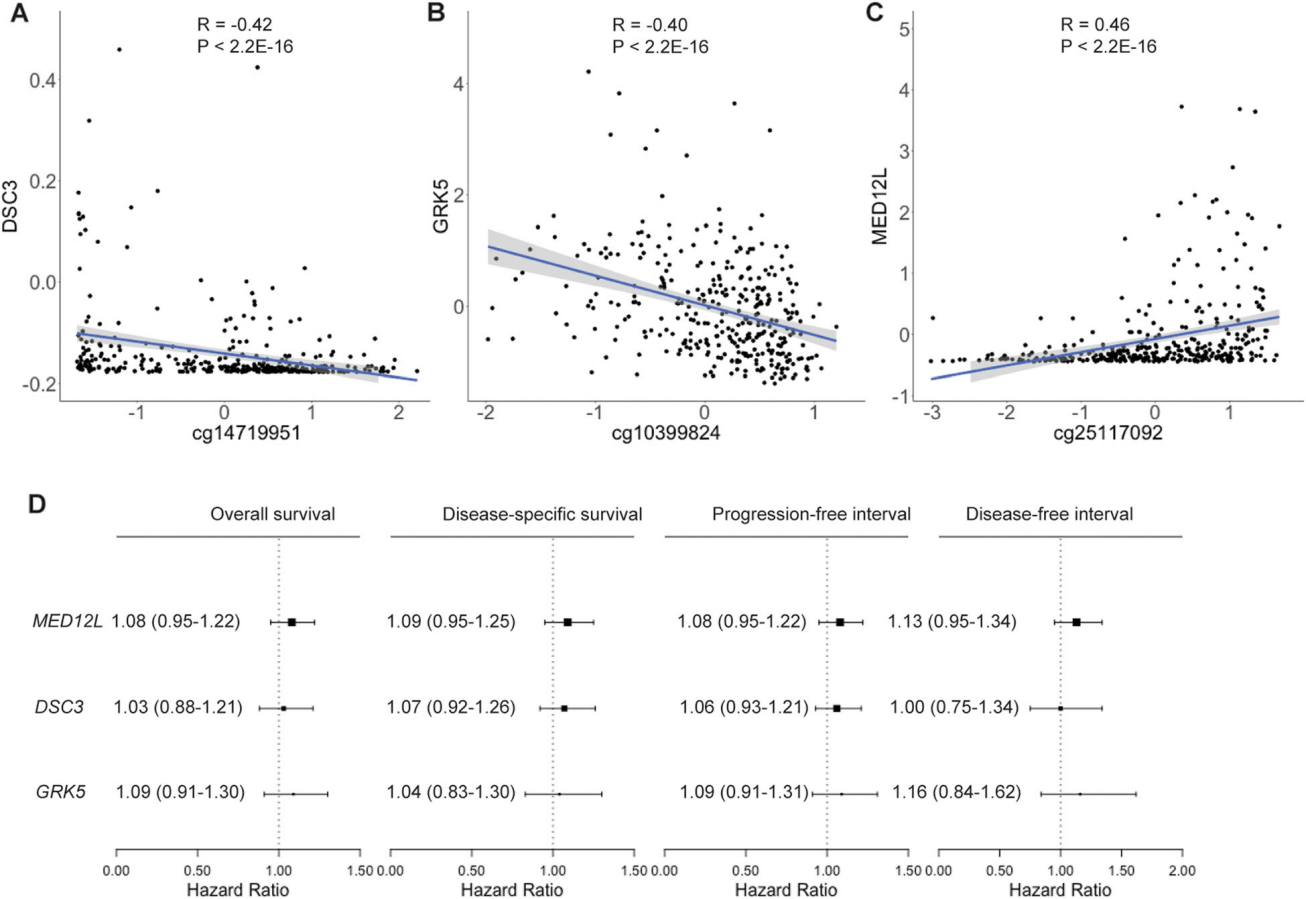
Spearman correlations between gene body DNA methylation of three CpG loci and host gene expression. **a** Spearman correlation for cg14719951 (*DSC3*). **b** Spearman correlation for cg10399824 (*GRK5*). **c** Spearman correlation for cg25117092 (*MED12L)*. **d** Forest plots indicating the association between mRNA expression of three genes and four clinical endpoints of GC patients

For the above highlighted key CpG sites in the gene body, we examined the associations of their DNA methylation level with host gene mRNA expression. Gene body DNA hyper-methylation of cg14719951 (correlation coefficient = − 0.42, *P*-value < 2.2 ×10^−16^) and cg10399824 (correlation coefficient = − 0.40, *P*-value < 2.2 ×10^−16^) was inversely associated with mRNA expression of *DSC3* and *GRK5*, respectively. On the contrary, Gene body DNA hyper-methylation of cg25117092 (correlation coefficient = 0.46, *P*-value < 2.2 ×10^−16^) was significantly associated with increased mRNA expression of *MED12L*. We did not find significant associations between the mRNA expression of these three genes and four clinical prognostic outcomes of GC (Fig. 3).

## Discussion

We comprehensively examined DNA methylation biomarkers associated with multiple prognostic outcomes of GC in an epigenome-wide association analysis based on TCGA. Altered DNA methylation of seven newly reported CpGs was significantly associated with OS, DSS and PFI of GC, including cg10399824 (*GRK5)*, cg05275153 (*RGS12)*, cg24406668 (*MMP9)*, cg14719951(*DSC3)*, and cg25117092 (*MED12L*), and two in intergenic regions (cg11348188 and cg11671115). Except for cg10399824 and cg24406668, five of them were also significantly associated with DFI of GC. Combining the significant CpG sites, two risk score models performed well in predicting GC prognosis. Consistent with individual CpG-level associations, DNA methylation of three MCBs involving cg11671115, cg14719951 and cg24406668 was significantly associated with multiple prognostic outcomes of GC.

Genomic location of DNA methylation sites is crucial for determining their potential biological functions [4, 24]. It is well known that aberrant promoter DNA hypermethylation could suppress gene expression [3]. The biological mechanisms of aberrant DNA methylation in intergenic regions or gene body, however, remained overall unclear [4, 25]. In the current study, cg11348188 and cg11671115 were located in intergenic regions and the other five highlighted CpG sites were located in gene body. Of them, DNA hypermethylation of *MED12L* was associated with increased mRNA expression, while hypermethylation of *DSC3* and *GRK5* were associated with decreased mRNA expression. Indeed, previous studies have also reported the complex associations between gene body hypermethylation and increased [4, 26] or decreased gene expression [25, 27]. The reason for the opposite directions of findings is unclear. Gene body DNA methylation might increase transcription through blocking the initiation of alternative promoters within gene body or regulating the activities of repetitive DNAs within the transcribed unit [24, 27]. In addition, splicing or elongation of the ordered structure within the transcriptional unit, which was induced by gene body DNA methylation, might also increase transcriptional activity [26]. However, intragenic regions also contain many functional elements, such as enhancers, transcription factor binding cites, and repetitive elements, activation of which induced by aberrant gene body DNA methylation could affect host gene expression. In our study, DNA hypermethylation of *RGS12* and *MMP9* were not correlated with host gene expression. Interestingly, previous studies on melanoma have reported gene body DNA hypermethylation and increased mRNA expression of *MMP9*, suggesting possible cell-type specificity of DNA methylation pattern [28, 29].

As aberrant promoter DNA hypermethylation could suppress gene expression, it would be essential to examine the location of five key CpGs in gene body region relative to their closest TSS, so that the effect of altered methylation on gene expression and GC prognosis can be interpreted reasonably. Except for cg1471995, the other four CpGs are located more than 1500 bp to the corresponding closest TSS, indicating that the gene body methylation of these four CpGs may be less likely to affect host gene expression as promoter. For cg1471995, as it was located on the intron 1 of *DSC3* with a distance of 308 bp to the closest TSS, it might act as the promoter regarding its effect on gene expression, which was consistent with our findings that high methylation level of cg14719951 was inversely associated with increased mRNA expression of *DSC3*.

We placed special interests on *MED12L*, *DSC3*, and *GRK5*, the DNA methylation of which was significantly associated with mRNA expression. Somatic mutations and DNA methylation may commonly target the same gene associated with cancer prognosis [30]. *MED12L* somatic mutations have been identified in both familial and sporadic GC as well as several other cancers [31, 32]. Promoter DNA hypermethylation of *DSC3*, which suppressed host gene expression, has been associated with poor OS of colorectal cancer and recurrence of prostate cancer [33, 34]. The potential role of *GRK5* DNA methylation in the progression/prognosis of cancer has been rarely studied. Interestingly, *MED12L*, *DSC3*, and *GRK5* mRNA expression was not associated with any GC prognostic outcome in our study. Therefore, gene body DNA methylation might affect GC prognosis not through regulating host gene expression, supporting that the identification of associations between DNA methylation and mRNA expression may not be prerequisites of developing DNA methylation-based biomarkers [4]. In fact, several commercially available DNA methylation-based biomarkers, including DNA methylation of *APC* (adenomatosis polyposis coli) and *RASSF1* (ras association domain family member 1) as both diagnostic and prognostic biomarkers of prostate cancer and DNA methylation of *SEPT9* (septin 9) as predictive biomarker of colorectal cancer, were not correlated with host gene expression [4].

Gene set enrichment analysis revealed that gene body methylation could affect GC prognosis by regulating the neuroactive ligand-receptor interaction pathway [35], which has been also enriched for a number of long noncoding RNAs associated with OS of GC in a prior study [36]. Moreover, this pathway has also been enriched for differentially methylated genes between tumor and normal tissue of GC [37], indicating a significant role in both carcinogenesis and prognosis of GC. Future studies are warranted to clarify how aberrant DNA methylation affect the neuroactive ligand-receptor interaction pathway.

Although DNA methylation of single CpG dinucleotides could effectively regulate host gene expression and are qualified as cancer prognostic biomarkers [38], the pertinent alterations of DNA methylation are often regional based, with interplays of a number of adjacent CpG sites. Therefore, in addition to identifying the optimal genomic location, defining the optimal number of relevant CpG sites are of great importance for developing DNA methylation-based biomarker assays [4]. In our study, we also examined DNA methylation patterns accounting for the cluster of adjacent CpG loci. The association of DNA methylation of three identified MCBs with GC prognosis was in line with that based on single CpG locus, which further strengthened our results.

Based on the publicly available dataset, our study was the first to thoroughly study DNA-methylation based biomarkers associated with multiple prognostic endpoints of GC, including OS, DSS, DFI, and PFI. The association between DNA methylation of seven key CpG loci and GC prognosis was newly reported. The genomic location and the optimal number of adjacent relevant CpG sites were critically evaluated and reported. Two DNA methylation-based risk score models performed well in predicting four clinical outcomes of GC patients. We were able to adjust for the major clinicopathological characteristics in sensitivity analyses, which did not change the results materially.

We acknowledged several limitations. First, DNA methylation profiles in TCGA were measured by Infinium 450 k microarrays, which might restrict us from identifying other uncovered CpG dinucleotides with biological relevance to GC prognosis. Second, although we tried to adjust for potential confounders, residual confounding might be present. Third, as an exploratory study, both discovery and validation datasets were utilized to identify prognostic CpG sites and were from the same dataset (TCGA), the performance of our risk score models might be inflated. External datasets with large sample size are warranted to further validate our results in the future. Fourth, our study was an association study based on biostatistical and bioinformatics analyses. Laboratory work is warranted to unravel the biological consequence of these types of DNA methylation.

In conclusion, our study newly identified seven CpG sites associated with OS, DSS, and PFI of GC, with five of them also associated with DFI of GC. Two DNA methylation-based risk score models were established, which may have implications for clinical practices regarding GC patient stratification of prognosis. Exploration in the experiment setting may contribute to our understanding of the underlying molecular mechanism of gene body DNA methylation on GC prognosis and may inspire the development of novel individualized DNA-methylation based therapeutic strategies.

## Supplementary Information

**Additional file 1: Supplementary Table S1.** Detailed information on gene set enrichment analyses for gene body DNA methylation in relation to four clinical endpoints of GC patients in TCGA.

## Data Availability

The datasets analyzed during the current study are available in the Genomic Data Commons Data Portal of National Cancer Institute (https://portal.gdc.cancer.gov/).
