# Supplementary material for: DNA methylation signatures associated with prognosis of gastric cancer
Source: BMC Cancer. 2021 May 25;21:610. doi: 10.1186/s12885-021-08389-0 (PMC8152126; doi:10.1186/s12885-021-08389-0)
Supplement: Supplementary file 1 — Additional file 1: Supplementary Table S1. Detailed information on gene set enrichment analyses for gene body DNA methylation in relation to four clinical endpoints of GC patients in TCGA. [file 12885_2021_8389_MOESM1_ESM.docx]

Supplementary Table S1. Detailed information on gene set enrichment analyses for gene body DNA methylation in relation to four clinical endpoints of GC patients in TCGA

| GO_ID | Description | Number of unique genes | Unique genes |
| --- | --- | --- | --- |
| GO_35267 | NuA4 histone acetyltransferase complex | 20 | *ACTB, ACTL6A, YEATS4, TRRAP, RUVBL1, MORF4L2, KAT5, RUVBL2, BRD8, MORF4L1, MSL3, EPC2, ACTL6B, ING3, MRGBP, DMAP1, EP400, MEAF6, EPC1, MSL3P1* |
| GO_36019 | Endolysosome | 20 | *AP2A1, AP2A2, AP2B1, AP2M1, AP2S1, CLTA, CLTC, CTSB, CTSK, CTSL, CTSS, HRG, LDLR, LGMN, TLR3, CLEC16A, TLR7, TLR8, TLR9, PCSK9* |
| GO_36020 | Endolysosome membrane | 14 | *AP2A1, AP2A2, AP2B1, AP2M1, AP2S1, CLTA, CLTC, LDLR, TLR3, CLEC16A, TLR7, TLR8, TLR9, PCSK9* |
| GO_43189 | H4/H2A histone acetyltransferase complex | 20 | *ACTB, ACTL6A, YEATS4, TRRAP, RUVBL1, MORF4L2, KAT5, RUVBL2, BRD8, MORF4L1, MSL3, EPC2, ACTL6B, ING3, MRGBP, DMAP1, EP400, MEAF6, EPC1, MSL3P1* |
| GO_44346 | Fibroblast apoptotic process | 24 | *BAK1, BID, BTG1, GAS6, MYC, PIK3CA, PIK3CG, SFRP1, TP53, XRCC2, CUL3, API5, TP63, STK17B, STK17A, BCL2L11, CFDP1, IER3IP1, PRDM11, CHD8, MIR181B1, MIR181B2, MIR24-1, MIR24-2* |
| KEGG_04080 | Neuroactive ligand-receptor interaction | 272 | *ADCYAP1R1, ADORA1, ADORA2A, ADORA2B, ADORA3, ADRA1D, ADRA1B, ADRA1A, ADRA2A, ADRA2B, ADRA2C, ADRB1, ADRB2, ADRB3, AGTR1, AGTR2, APLNR, AVPR1A, AVPR1B, AVPR2, BDKRB1, BDKRB2, BRS3, TSPO, C3AR1, C5AR1, CALCR, CCKAR, CCKBR, CGA, CHRM1, CHRM2, CHRM3, CHRM4, CHRM5, CHRNA1, CHRNA2, CHRNA3, CHRNA4, CHRNA5, CHRNA7, CHRNB1, CHRNB2, CHRNB3, CHRNB4, CHRND, CHRNE, CHRNG, LTB4R, CNR1, CNR2, CRHR1, CRHR2, CSH1, CTSG, DRD1, DRD2, DRD3, DRD4, DRD5, S1PR1, LPAR1, S1PR3, EDNRA, EDNRB, F2, F2R, F2RL1, F2RL2, FPR1, FPR2, FPR3, FSHB, FSHR, GABBR1, GABRA1, GABRA2, GABRA3, GABRA4, GABRA5, GABRA6, GABRB1, GABRB2, GABRB3, GABRD, GABRE, GABRG1, GABRG2, GABRG3, GABRP, GABRR1, GABRR2, GALR1, GCGR, GH1, GH2, GHR, GHRHR, GHSR, GIPR, GLP1R, GLRA1, GLRA2, GLRB, GNRHR, NPBWR1, NPBWR2, PRLHR, UTS2R, LPAR4, MCHR1, GPR35, MLNR, GRIA1, GRIA2, GRIA3, GRIA4, GRID1, GRID2, GRIK1, GRIK2, GRIK3, GRIK4, GRIK5, GRIN1, GRIN2A, GRIN2B, GRIN2C, GRIN2D, NR3C1, GRM1, GRM2, GRM3, GRM4, GRM5, GRM6, GRM7, GRM8, GRPR, GZMA, HCRTR1, HCRTR2, HRH1, HRH2, HTR1A, HTR1B, HTR1D, HTR1E, HTR1F, HTR2A, HTR2B, HTR2C, HTR4, HTR5A, HTR6, HTR7, LEP, LEPR, LHB, LHCGR, MAS1, MC1R, MC2R, MC3R, MC4R, MC5R, MTNR1A, MTNR1B, NMBR, NPY1R, NPY2R, NPY5R, NTSR1, OPRD1, OPRK1, OPRL1, OPRM1, OXTR, P2RX1, P2RX3, P2RX4, P2RX5, P2RX7, P2RY1, P2RY2, P2RY4, P2RY6, P2RY11, PLG, NPY4R, PRL, PRLR, PRSS1, PRSS2, PRSS3, PTAFR, PTGDR, PTGER1, PTGER2, PTGER3, PTGER4, PTGFR, PTGIR, PTH1R, PTH2R, SCTR, SSTR1, SSTR2, SSTR3, SSTR4, SSTR5, TACR2, TACR1, TACR3, TBXA2R, THRA, THRB, TRHR, TSHB, TSHR, VIPR1, VIPR2, TRPV1, GLRA3, GALR3, S1PR4, GALR2, CHRNA6, F2RL3, TAAR5, P2RX6, LPAR2, GPR50, TAAR2, S1PR2, GLP2R, GABBR2, P2RY14, LPAR6, CALCRL, NMUR1, CYSLTR1, NPFFR2, GPR83, HRH3, P2RX2, LPAR3, NTSR2, P2RY10, S1PR5, P2RY13, CHRNA9, GABRQ, PARD3, LTB4R2, NMUR2, CHRNA10, CYSLTR2, HRH4, RXFP1, NPFFR1, TAAR8, MCHR2, KISS1R, GRIN3A, GRIN3B, RXFP2, TAAR9, TAAR1, GPR156, P2RY8, TAAR6* |
| KEGG_04144 | Endocytosis | 201 | *ADRB1, ADRB2, ADRB3, GRK2, GRK3, AP2A1, AP2A2, AP2B1, ARF6, RHOA, ARRB1, ARRB2, CAV1, CAV2, CAV3, CBL, CBLB, CDC42, AP2M1, AP2S1, CLTA, CLTB, CLTC, CCR5, CSF1R, DAB2, DNM1, DNM2, EGF, EGFR, EPS15, ERBB3, ERBB4, F2R, FGFR3, FGFR2, FGFR4, FLT1, FOLR1, FOLR2, FOLR3, GRK4, GRK5, GRK6, HLA-A, HLA-B, HLA-C, HLA-E, HLA-F, HLA-G, HRAS, HSPA1A, HSPA1B, HSPA1L, HSPA2, HSPA6, HSPA8, IGF1R, IL2RA, IL2RB, IL2RG, CXCR1, CXCR2, KDR, KIT, LDLR, SMAD2, SMAD3, SMAD6, SMAD7, MDM2, MET, NEDD4, NTRK1, PDGFRA, PLD1, PLD2, PML, PRKCI, PRKCZ, PSD, RAB4A, RAB5A, RAB5B, RAB5C, RET, GRK1, SH3GL1, SH3GL2, SH3GL3, SRC, TFRC, TGFB1, TGFB2, TGFB3, TGFBR1, TGFBR2, TRAF6, TSG101, CXCR4, RAB7A, STAM, CLTCL1, PIP5K1A, PIP5K1B, EEA1, RAB11A, ASAP2, USP8, RABEP1, HGS, RAB11B, ZFYVE9, VPS4B, RAB11FIP3, ACAP1, ZFYVE16, GIT2, DNAJC6, IQSEC1, PDCD6IP, DNM1L, RNF41, STAM2, STAMBP, EHD1, RAB31, WWP1, SNF8, VPS45, RAB11FIP2, EPN2, IQSEC2, NEDD4L, PSD3, PIP5K1C, ACAP2, PSD4, CBLC, CHMP2B, DNM3, RAB11FIP5, LDLRAP1, ARFGAP3, VPS4A, CHMP2A, GIT1, CHMP4A, EPN1, SH3KBP1, EHD4, EHD3, EHD2, ASAP1, PARD6A, VPS36, SH3GLB1, VPS28, CHMP5, VTA1, CHMP3, EPN3, VPS37C, ASAP3, ARFGAP1, PARD3, SH3GLB2, CHMP1B, SMURF1, RAB22A, SMAP1, RBSN, ARAP3, SMAP2, SMURF2, CHMP6, VPS37B, RAB11FIP1, RUFY1, ITCH, PSD2, VPS25, ARFGAP2, RAB11FIP4, PARD6G, PARD6B, MVB12B, CHMP4C, MVB12A, ACAP3, ARAP2, ARAP1, AGAP2, AGAP1, CHMP4B, GRK7, VPS37A, PIP5KL1, VPS37D, IZUMO1R, IQSEC3* |
| KEGG_04950 | Maturity onset diabetes of the young | 25 | *NR5A2, GCK, HHEX, MNX1, FOXA2, FOXA3, HNF4A, HNF4G, ONECUT1, HES1, IAPP, INS, PDX1, NEUROD1, NKX2-2, NKX6-1, PAX4, PAX6, PKLR, SLC2A2, HNF1A, HNF1B, NEUROG3, BHLHA15, MAFA* |
| KEGG_05217 | Basal cell carcinoma | 55 | *APC, BMP2, BMP4, CTNNB1, DVL1, DVL2, DVL3, FZD2, GLI1, GLI2, GLI3, GSK3B, PTCH1, SHH, SMO, TCF7, TCF7L2, TP53, WNT1, WNT2, WNT3, WNT5A, WNT6, WNT7A, WNT7B, WNT8A, WNT8B, WNT10B, WNT11, WNT2B, WNT9A, WNT9B, FZD5, FZD3, AXIN1, AXIN2, FZD1, FZD4, FZD6, FZD7, FZD8, FZD9, PTCH2, APC2, FZD10, STK36, LEF1, WNT16, SUFU, WNT4, HHIP, WNT10A, WNT5B, TCF7L1, WNT3A* |
